# Cecal Volvulus

**DOI:** 10.5334/jbsr.2766

**Published:** 2022-05-04

**Authors:** Kelly Di Dier, Adelard De Backer, Filip Vanhoenacker

**Affiliations:** 1 AZ Sint-Lucas, BE; 2 AZ Sint-Maarten, BE

**Keywords:** abdominal computed tomography, cecal volvulus, whirl sign, bird’s beak sign, coffee bean sign

## Abstract

**Teaching Point:** The coffee bean sign, the whirl sign, and the bird’s beak sign are the key findings on abdominal CT of cecal volvulus.

## Case History

An 81-year-old woman presented at the emergency department with severe progressive abdominal pain and vomiting for two days. Clinical examination showed abdominal tenderness without rebound and decreased peristalsis.

**Figure 1:**
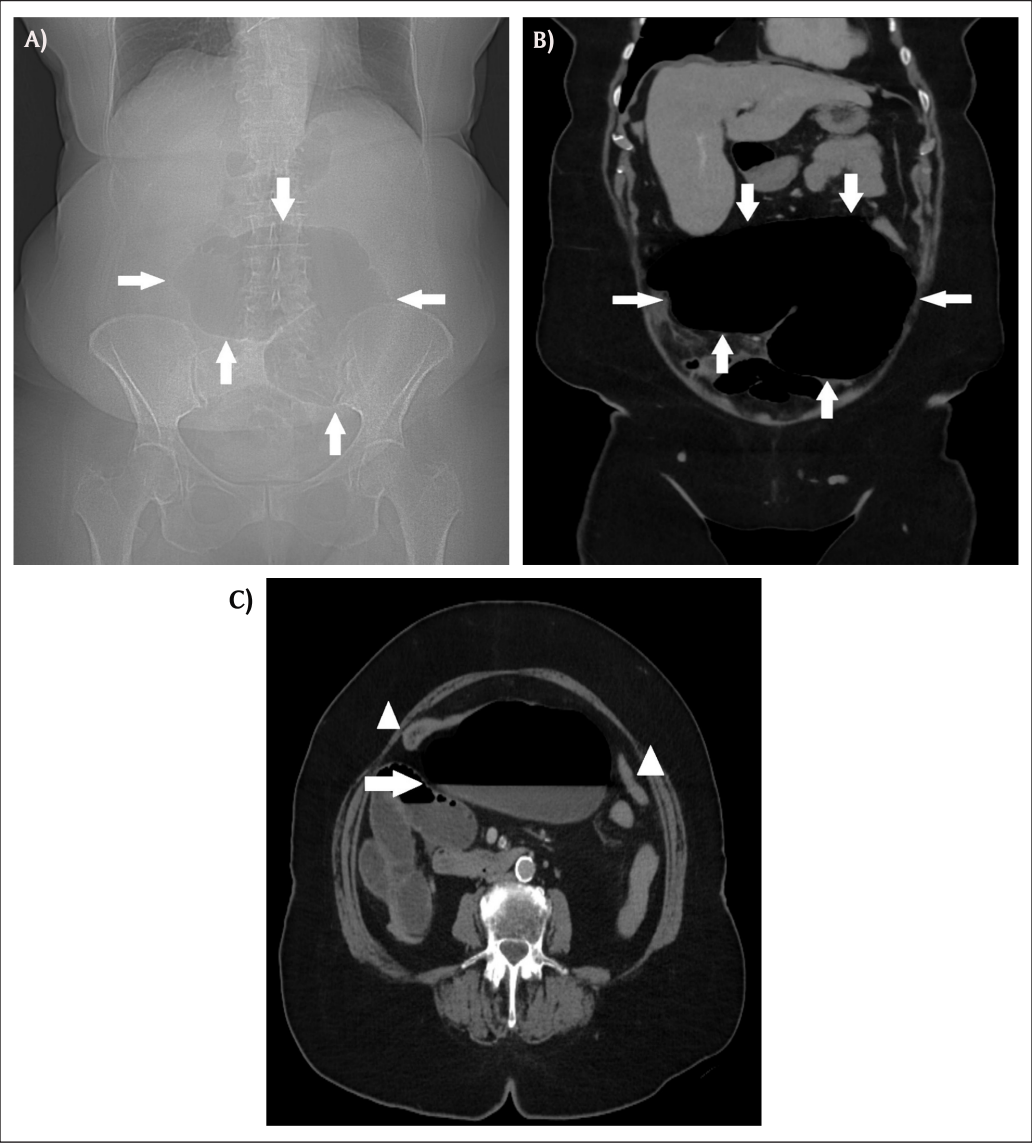


**Figure 2:**
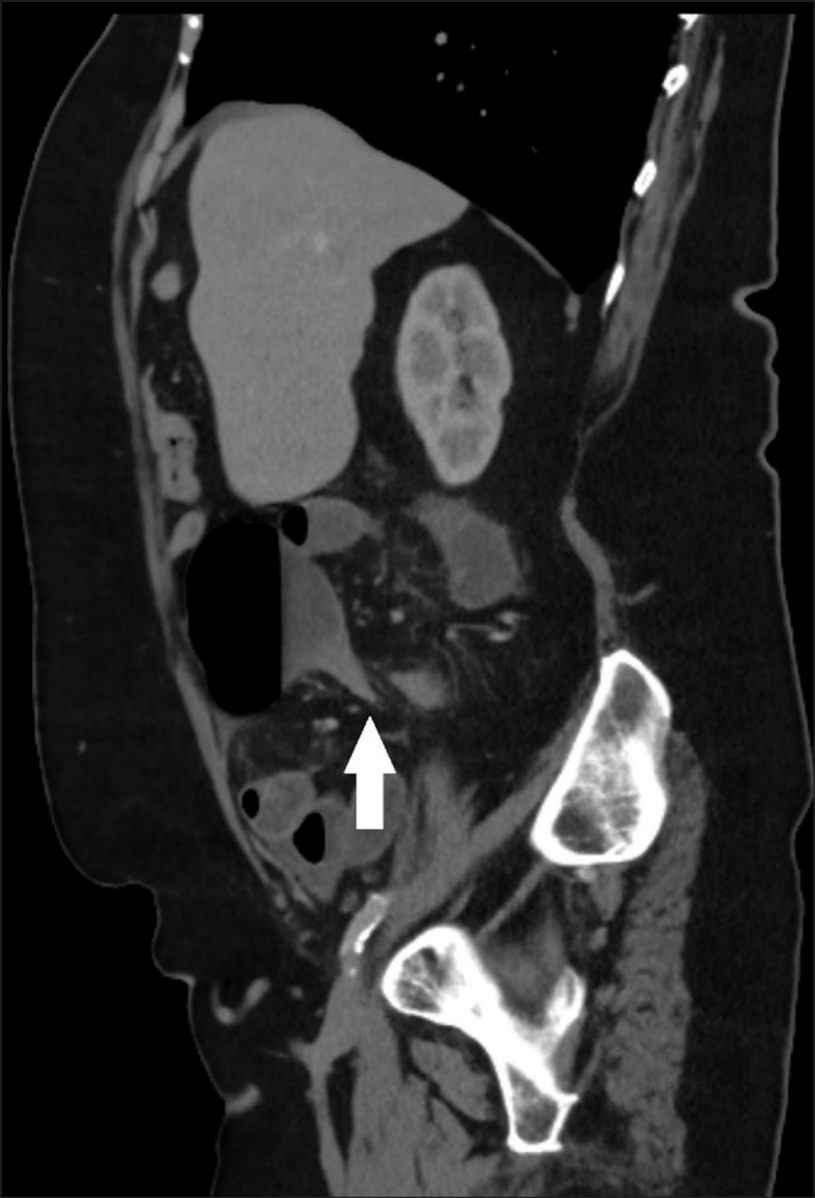


Scout view of abdominal computed tomography (CT) showed an air-distended bowel loop in the lower abdomen with the shape of a coffee bean (***Figure 1A***, arrows). Coronal contrast-enhanced CT showed a distended cecum measuring 9 centimeters (***Figure 1B***, arrows), with air-fluid level (***Figure 1C***, arrow) and collapse of the transverse and left colon on axial images (***Figure 1C***, arrowheads). The coronal images also showed a clockwise whirl of spiraling collapsed cecum; fatty mesentery with enhancing engorged vessels and a central soft-tissue density was noted (***Supplementary video***). On sagittal reformatted images, gradual tapering of the wall of the obstructed cecum at the site of the whirl resulted in a stenosis with the appearance of a bird’s beak (***Figure 2***). The distal ileum was fluid-filled. There were no signs of bowel perforation. The presumed diagnosis of closed loop obstruction due to cecal volvulus was confirmed by laparoscopy. Right hemicolectomy was performed.

**Video:** https://youtu.be/4zsE3ySqktQ

## Comment

Volvulus occurs when a fixed intestinal base is followed by a loose twisted segment, resulting in a closed-loop obstruction. When the colon is affected, sigmoid volvulus is most often seen. Cecal involvement is rather rare. Cecal volvulus may occur in patients with a developmental failure of peritoneal fixation, resulting in a mobile proximal colon. Whilst sigmoid volvulus is common among the elderly, cecal volvulus occurs in younger patients generally between 30–60 years [1].

Typical symptoms include various degrees of acute cramping abdominal pain, nausea, and vomiting. Findings on clinical examination may vary and generally will not contribute to the final diagnosis [1].

Abdominal CT is crucial to make a correct diagnosis. Torsion of the cecum may rotate clockwise or counterclockwise in the axial plane around its long axis. If present, the obstructed cecum will appear in the right lower quadrant. An extreme air-distended cecum with visible haustral folds may resemble a coffee bean on scout view, axial, or coronal CT images. A whirl sign presents a centrally located twisted cecum surrounded by swirling mesenteric vessels and fat. A central soft-tissue density pinpoints the source of the twist. A bird’s beak sign results from gradual tapering of the cecum at the site of torsion, resulting in the appearance of a bird’s beak [1].

The treatment is mainly surgical. If uncomplicated, detorsion with cecopexy may be performed to prevent recurrence. If complicated (e.g., bowel ischemia, necrosis, or perforation) right hemicolectomy is mandatory [1].
